# Investigation of the mental health and cognitive correlates of psychological decentering in adolescence

**DOI:** 10.1080/02699931.2024.2402947

**Published:** 2024-10-02

**Authors:** R. C. Knight, D. L. Dunning, J. Cotton, G. Franckel, S. P. Ahmed, S. J. Blakemore, T. Ford, W. Kuyken, MYRIAD Team, T. Dalgleish, M. P. Bennett

**Affiliations:** a MRC Cognition and Brain Sciences Unit, University of Cambridge, Cambridge, UK; b Department of Health Sciences, University of York, York, UK; c Institute of Cognitive Neuroscience, University College London, London, UK; d Department of Psychology, University of Cambridge, Cambridge, UK; e Department of Psychiatry, University of Cambridge, Cambridge, UK; f Department of Psychiatry, University of Oxford, Oxford, UK

**Keywords:** Decentering, adolescence, mental health, psychological decentering, cognitive control

## Abstract

The ability to notice and reflect on distressing internal experiences from an objective perspective, often called psychological decentering, has been posited to be protective against mental health difficulties. However, little is known about how this skill relates to age across adolescence, its relationship with mental health, and how it may impact key domains such as affective executive control and social cognition. This study analysed a pre-existing dataset including mental health measures and cognitive tasks, administered to adolescents in Greater London and Cambridge (mean age (SD) = 14.4 (1.77) years, *N* = 553). A self-report index of decentering based on available questionnaire items in the dataset was developed. Multiple linear regression was used to examine associations between decentering and mental health, affective executive control (measured using an affective Stroop Task, affective Working Memory Task, and affective Sustained Attention to Response Task) and social cognition. Higher decentering was significantly associated with lower depression and anxiety scores and higher psychological wellbeing. Results did not indicate significant relationships between decentering, affective executive control and social cognition. Further research is needed to discover cognitive mechanisms associated with this process, which could allow for optimisation of existing psychological therapy and reveal new avenues of intervention.

## Introduction

A feature across mental health difficulties is the triggering of challenging thoughts, feelings and memories by socio-environmental cues. Behavioural patterns then develop, typically consistent with the content of these mental experiences (e.g. I try to escape when danger is expected). Importantly, people have capacity to not only react to cues but also reflect on, and interpret, what is happening mentally during emotionally taxing moments (e.g. I’m having a thought that this situation is dangerous). This self-reflective activity gives an opportunity to mentally “step back” – so one might respond to cues with greater flexibility than if mental experiences were treated like precise reflections of how the world really is. We refer to this self-reflective activity as psychological decentering, a strategy that reduces the affective intensity of internal experiences by allowing individuals to expand their initial awareness (Safran & Segal, 1996).

In a recent review, we described psychological decentering as a skill reinforced in many psychological interventions (Bennett et al., 2021; Bernstein et al., 2015; Hayes-Skelton & Lee, 2018). Cognitive defusion in acceptance-based treatments, decentering in mindfulness-based therapies, the observer self in psychoanalytic treatments and self-distancing in cognitive therapies, all exercise the capacity to generate new self-perspective by bringing attentional focus to mental experiences. Emerging theories suggest that psychological decentering and related constructs are underpinned by well-understood executive functions. Powers and LaBar specifically suggested that a cascade of cognitive mechanisms plays a role in how individuals reflect on, and re-appraise, their internal mental experiences (Powers & LaBar, 2019). A goal is mentally formulated – namely, a less intense emotional state – and then maintained in attention and working memory. A process of active self-reflection next allows an individual to identify the emotion they wish to diminish. Self-projection is also used to mentally simulate oneself in a different space, drawing on elements of self-relevant information, including prior memories and knowledge. There is limited evidence for an association between psychological decentering and these executive functions (Kessel et al., 2016). However, prior research has established a mediating effect of adaptive or maladaptive emotion regulation strategies on executive functioning, measured using a parent-report scale, in early adolescence (Wante et al., 2017).

Adolescence is a critical developmental stage for research on psychological decentering and mental health. Adolescence is firstly a key period for the development of symptoms that are typically reduced by psychological decentering based interventions, including anxiety and depression (Hayes-Skelton & Lee, 2018; Kessel et al., 2016). However, it is also a time of marked change in executive functions that may underlie the impact psychological decentering has on emotion, such as affective cognitive control and response inhibition. Yet little is known about the relationship between early emerging symptoms and the capacity to reflect on internal mental states in the context of this developmentally important period. Lastly, adolescence is a significant time of social and interpersonal flux that can impact on patterns of thinking and behaviour. This is seen, for example, through the influence that peers have on a young person’s thinking and behaviour, which reaches its peak during adolescence (Ahmed et al., 2020; Steinberg & Monahan, 2007). Social influence can be both negative (e.g. antisocial behaviour) and positive (e.g. emergent altruism) but little is known about factors that might impact one’s susceptibility to such complex interpersonal dynamics. It could be, for example, that greater capacity for self-reflection and awareness mitigates one’s susceptibility to social influence. Questions that require exploration include: (1) does decentering differ as a function of age and sex during adolescence, (2) how does decentering relate to symptoms of poor mental health and (3) how is decentering linked with two key developmental domains: executive functioning and social cognition.

A systematic review conducted by our research team investigated the relationship between youth mental health (12-18-year-olds) and the tendency to overly identify with thoughts and feelings – measured using an inventory known as the Youth Avoidance and Fusion Questionnaire (Y-AFQ) (Bennett et al., 2021). The Y-AFQ is a self-report assessment in which higher scores indicate a tendency to buy into the content of internal mental experiences. Findings suggested a moderate-strong association between Y-AFQ scores and the severity of symptoms of anxiety and depression (Bennett et al., 2021). However, this study investigated the activity of becoming fused with thoughts, rather than stepping back from them. Further research, which measures psychological decentering rather than peripheral, or even opposing, constructs such as cognitive fusion, is needed.

Few studies have investigated how psychological decentering is associated with executive control and social cognition. One study used a Stroop task, which estimates executive function by comparing response times to incongruent (e.g. the word red in blue ink) relative to congruent stimuli (e.g. the word red in red ink). This task was administered to participants after watching aversive film clips under different conditions; one used a therapeutic strategy to prompt awareness and reflection on one’s thoughts; and one used a control condition with no instructed strategy (Pilecki & McKay, 2012). Results indicated that reflecting on thoughts facilitated quicker responses to colour incongruent stimuli, compared with no strategy. This suggests emotional distress interferes with executive functioning, but this effect is minimised when awareness of distress-related thoughts, or decentered self-perspective, is prompted. Fewer studies still have investigated how social changes during adolescence impact capacity to reflect on internal mental states to generate meaning. Available findings, however, suggest a negative association between psychological distancing and social distress such as exclusion-related pain and performance anxiety (Barrera et al., 2016; Yanagisawa et al., 2011).

The current study had several aims and hypotheses. The first was to investigate the association between psychological decentering, and mental health and wellbeing in an adolescent sample. It was expected that the capacity to be aware of, and reflect on, internal mental states – estimated using a self-report measure of decentering – would reduce the risk of depression and anxiety, and improve wellbeing (Hypotheses 1a and 1b). A second aim was to examine the association between decentering and performance-based measures of affective executive control. Affective interference on task performance was expected to be lower in those with greater ability to adopt a decentered self-perspective. This would be characterised by individuals reporting higher decentering showing, in emotion contexts, more accurate recall of verbal information (Hypothesis 2), more sustained attention (Hypothesis 3) and cognitive control (Hypothesis 4). A final aim was to explore the relationship between psychological decentering and social cognition. We specifically hypothesised that capacity to decenter might reduce the influence of peers on decision making and behaviour (Hypothesis 5). Hypotheses, methods and analysis strategies were pre-registered on Open Science Framework. (https://osf.io/2q4t5).

## Method

### Participants, datasets and ethics

The MYRIAD (www.osf.io/d6y9q) dataset was collected as part of a trial investigating the mental health and cognitive outcomes of a school-based mindfulness training programme. Participants were recruited from 12 secondary schools in Cambridge and Greater London (*N* = 553, 31.53% male, mean age 14.41 (1.77) years). The present study analysed baseline data (Time 1) collected prior to randomisation to the treatment condition.

### Measures and materials

Full details of the mental health inventories and tasks of executive functioning and social cognition collected in the MYRIAD trial are reported elsewhere (Dunning et al., 2022; Griffiths et al., 2022; Leung et al., 2023). A summary of measures used in this study is provided below.

#### Decentering measure

A self-rated measure of psychological decentering was developed using available items from two emotional regulation questionnaires – the Difficulties in Emotion Regulation Scale (DERS) and the Child and Adolescent Mindfulness Measure (CAMM) (Gratz & Roemer, 2004; Greco et al., 2011). To create this measure, available items in the DERS and the CAMM – referred to as “candidate items” – were matched to items from pre-existing psychological decentering inventories, which described an ability to reflect on and interpret mental experiences from an objective self-perspective (Appendix A). For example, the candidate item “*I get upset with myself for having certain thoughts*”, taken from the CAMM, was matched with the item “*My anxious thoughts and feelings are not normal*” taken from the Believability of Anxious Feelings and Thoughts scale (Herzberg et al., 2012). This process was based on methods developed by Cotton & Baker (Cotton & Baker, 2019). Full details of measure creation are available in Supplemental Materials and Appendix B–C.

#### Mental health measures

Depression symptoms were measured using the Centre for Epidemiological Studies – Depression Scale (CES-D; (Radloff, 1977)). Anxiety symptoms were measured using the Revised Child Anxiety and Depression Scale – Anxiety Subscale (RCADS; (Ebesutani et al., 2012)), and psychological wellbeing was measured using the Warwick–Edinburgh Mental Wellbeing Scale (WEMWBS; (Tennant et al., 2007)).

#### Executive functioning tasks

Three affective executive functioning tasks were included from the MYRIAD dataset.

##### Emotional working memory task (eWMT)

Participants were asked to count shapes, memorize words and then report them back. A negative background image was present on one half of trials (e.g. an image showing social exclusion) and a neutral image was present on the other half (e.g. an object like a ball). The dependent variable was the proportion of words correctly recalled in neutral blocks minus negative blocks.

##### Affective sustained attention to response task (aSART)

Participants were required to press the space bar each time a number appeared, with the exception of the number three, where participants were instructed to withhold a response. Trials either had negative (e.g. babies crying) or neutral sounds (e.g. birdsong) playing during. The dependent variables were a number of commission errors and reaction time variance. Performance during neutral blocks was subtracted from negative blocks.

##### Emotional stroop task

A series of faces with either happy, neutral or sad expressions were shown, along with an adjective describing a happy or sad emotional state (e.g. joyful, upset). Face and word pairs were either emotionally congruent (matching expression and word), emotionally incongruent (e.g. sad expression and happy adjective), or neutral (any stimulus where the face was neutral). Participants categorised the affective adjectives as either happy or sad whilst ignoring the facial expression valence. The dependent variable was the difference in reaction time between congruent and incongruent trials.

#### Social cognition

##### Social influence task

This task assessed prosocial and antisocial behaviours, and the degree to which participants were influenced by others reported behaviours. Participants were presented with prosocial and antisocial behaviours and asked to rate how often they engage in them. Participants were then shown a rating of the same behaviour, which was reportedly the average answer provided by other adolescents and then asked to rate the same behaviour again. The dependent variable was the difference between participants’ initial ratings and ratings after seeing “average” ratings.

##### Prosocial giving task

This task assessed participants’ altruistic prosocial behaviour by asking them in a single question how they would split a sum of money (£5) between themselves and a charity, selected from a choice of five charities. The dependent variable was the percentage of money they chose to give to charity.

### Analysis strategy

The association between decentering and each of the following: demographic factors of age and sex, mental health measures, social cognition and affective cognitive control tasks were investigated using multiple linear regression.^1^ A Bonferroni correction was used to correct for multiple comparisons within each group of analyses. Data analyses were completed using RStudio (V1.3.1093) and SPSS (28.0.0.0 (190)). The full analysis strategy can be found on OSF.

## Results

### Measure of decentering

The final measure of psychological decentering had 11 items, with scores ranging from 11 to 55 – mean and SD in Table 1. The scale had good internal consistency (Cronbach’s *α* = .87). Factor analysis indicated that four items should be removed from this scale. The removal of these items did not impact the outcome of planned analysis analyses. We report findings based on an 11-item version of the scale. Information on the 7-item version of the scale can be found in Supplemental Materials.

**Table 1.** Means and standard deviations of questionnaire and task data.

			*M*	*SD*
Demographics				
	Age		14.41	1.770
Questionnaires				
	Self-rated decentering		39.83	8.707
	Depression (CES-D)		17.26	10.10
	Anxiety (RCADS)		12.92	7.781
	Wellbeing (WEMWBS)		46.81	9.382
Tasks				
	Emotional working memory task (eWMT)		−1.377	11.64
	Affective sustained attention to response task (aSART)	Commission errors	0.426	3.606
		Reaction time variance	0.012	0.079
	Emotional stroop task	Negative vs positive	37.44	104.5
		Negative vs neutral	−30.03	109.8
		Neutral vs positive	7.415	112.9
	Social influence task		0.778	0.497
	Prosocial giving task		0.638	0.264

### Does decentering differ across age and sex?

**Figure 1.**
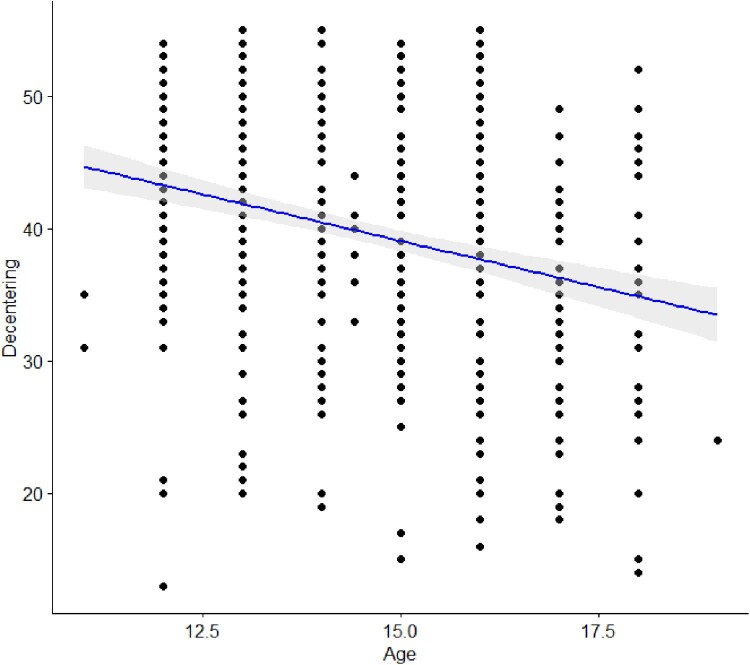
Scatter plot of association between age and decentering.

Supplemental to pre-registered analyses, a linear regression was calculated to test whether decentering scores were predicted by age and sex, simultaneously. This model accounted for 8.3% of the variance in decentering score (Adj *R*^2^ = 0.083, *F*(2,548) = 25.92, *p* < 0.001, *f*^2 ^= 0.09). Results indicated that decentering scores were significantly associated with sex (*β* = 0.250, *p *< .001) with males reporting higher scores than females. Also, decentering scores decreased as age increased across adolescence (*β* = −0.253, *p *< .001), (see Figure 1).

A hierarchical model was calculated to investigate whether these effects remained after including other potential covariates of decentering in the model. Step 1 included depression symptoms, anxiety symptoms and wellbeing scores to predict decentering scores. This model accounted for 55% of the variance (Adj *R*^2^ = 0.546, *F*(3,549) = 222.3, *p* < 0.001, *f*^2 ^= 1.20). Depression (β = −0.399, *p *< .001), anxiety (*β* = −0.357, *p *< .001) significantly predicated decentering, and wellbeing scores did not (*β* = 0.078, *p *> .05). Step 2 added age as a predictor. Age significantly predicted decentering scores after accounting for covariates (*β* = −0.186, *p *< .001), with the age included model accounting for 58% of the variance in decentering scores (Adj *R*^2^ = 0.581, *F*(4,548) = 190.3, *p* < 0.001, *f*^2 ^= 1.37). Step 3 added sex as a predictor variable. Sex did not significantly predict decentering scores after accounting for effects of covariates and age.

### Does decentering relate to mental health?

Means and standard deviations for each mental health measure are available in Table 1. Multiple regression analysis was used to test if decentering, age and sex significantly predicted self-rated (i) depressive, (ii) anxiety symptoms and (iii) psychological wellbeing. A Bonferroni correction was used to account for multiple comparisons, with an adjusted significance threshold of 0.0167.

#### Depressive symptoms

A multiple linear model was calculated to test whether depression symptoms were predicted by decentering scores after adjusting for effects of age and sex. This model accounted for 46.1% of the variance in self-rated depression (Adj *R*^2^ = 0.461 *F*(3,547) = 157.8, *p* < 0.001, *f*^2 ^= 0.85). As predicted, self-reported psychological decentering was significantly negatively associated with depression symptoms (*β* = −0.662, *p* < .001). That is, higher self-reported decentering predicts lower depressive symptoms. Decentering was also significantly associated with sex, such that being female predicted worse depression symptoms (*β* = −0.211, *p *< .01).

#### Anxiety symptoms

A multiple linear model was calculated to test whether anxiety symptoms were predicted by decentering scores after adjusting for effects of age and sex. This model accounted for 44.3% of the variance in self-rated anxiety (Adj *R*^2^ = 0.444, *F*(3,547) = 147.1, *p* < 0.001, *f*^2 ^= 0.80). Psychological decentering was negatively associated with anxious symptoms (*β* = −0.673, *p *< .01). Anxiety was also significantly associated with sex (*β* = −0.220, *p *< .001), and age (*β* = −0.175, *p *< .001), such that being older and female predicted higher anxiety.

#### Wellbeing

A multiple linear model was calculated to test whether wellbeing was predicted by decentering scores after controlling for effects of age and sex. This model accounted for 30.7% of the variance in self-rated wellbeing (Adjusted *R*^2^ = .307, *F*(3,547) = 82.25, *p* < 0.001, *f*^2 ^= 0.44). Decentering was significantly associated with wellbeing (*β* = 0.540, *p *< .001), such that higher self-reported decentering predicts higher self-reported wellbeing.

#### Hierarchical model assessing independence of decentering as a predictor of wellbeing

Supplemental to pre-registered analysis, a hierarchical multiple regression was calculated to test if the association of decentering with wellbeing was independent of depression and anxiety symptoms. This step was included because of the co-varience between these variables, details of which can be found in Supplemental Materials.

Step 1 included age as a predictor of wellbeing. This model revealed a significant association between age and wellbeing scores (Adjusted *R*^2^ = 0.286, *F*(1,551) = 17.24, *p* < 0.001, *f*^2^ = 0.04). Step 2 included age and sex. This improved the model accuracy (Adjusted *R*^2^ = 0.414, *F*(2,548) = 12.88, *p* < 0.001, *f*^2^ = 0.07). Step 3 included depression and anxiety scores. This significantly improved the model accuracy (Adjusted *R*^2^ = 0.555, *F*(4,546) = 172.2, *p* < 0.001, *f*^2^ = 1.24); however, anxiety symptoms were not a significant predictor (*β = −*0.046, *p* = 0.218). After addition of depression and anxiety scores, sex and age were also no longer significant predictors. In step 4, decentering was added as a predictor. Decentering was not a significant predictor of wellbeing (*β = *0.066, *p* = 0.134). This suggests that the association of decentering with psychological wellbeing is not independent of depression severity.

### Does decentering relate to executive functioning and social cognition?

#### Decentering and affective executive control

Means and standard deviations of variables from the eWMT, affective SART, and Emotional Stroop Task are available in Table 1. Linear regression models were performed to ascertain whether decentering predicted each task variable and corrected for multiple comparisons. These models did not pass the threshold for statistical significance before correction (Table 2).

**Table 2.** Linear regression models predicting task scores using decentering scores.

Task	Model	*R*^2^	*F*	*p*	*f*^2^
eWMT	Decentering * eWM index	0.000	0.949	0.331	−0.00
aSART	Decentering * commission errors	0.001	1.764	0.187	0.00
	Decentering * reaction time variance	0.003	1.534	0.216	0.00
Stroop task	Decentering * negative and neutral diff	−0.001	0.578	0.448	−0.00
	Decentering * positive and neutral Diff	0.001	1.396	0.238	0.00
	Decentering * positive and negative diff	−0.001	0.229	0.633	−0.00
Social influence	Decentering * absolute rating difference	−0.001	0.386	0.535	−0.00
Prosocial giving	Decentering * percentage donated	−0.002	0.002	0.966	−0.00

*df* = 1551.

#### Decentering and social cognition

Means and standard deviations of variables from both the social tasks are available in Table 1. Linear regression models were calculated to examine whether psychological decentering predicted performance on either social cognition task and corrected for multiple comparisons. Neither regression model reached significance before correction (Table 2).

## Discussion

The capacity to self-reflect and notice what’s on one’s mind in emotional situations is an important one. Mentally “stepping back” can allow for a broader self-perspective and offer space for more flexible response patterns. We call this capacity psychological decentering. However, it is unclear whether this capacity contributes to mental health and wider domains during adolescence, a critical window for the onset of mental health difficulties and socio-cognitive change. This study analysed an existing dataset to examine the association between self-rated psychological decentering and mental health, and two key developmental domains – executive control and social cognition.

Findings supported our first hypotheses. Depression and anxiety symptoms were fewer, and wellbeing was higher, in adolescents who reported a greater ability to adopt a decentered self-perspective. It may be that the association between this decentered perspective and wellbeing was indirectly driven by depression and anxiety. Hierarchical regression, for example, indicated that psychological decentering scores were no longer associated with wellbeing after co-varying depression and anxiety scores. This suggests that decentering, although related to symptoms of common emotional difficulties, may not improve wellbeing over and above reductions in symptoms. Overall, these results are consistent with evidence from older adolescents and young adults, that the ability to take an objective self-perspective is associated with better wellbeing and lower mental health symptomatology (see Bennett et al., 2021 for review).

Our second set of hypotheses predicted that the ability to notice internal experiences and step back would correlate with aspects of affective executive functioning, as measured via standard performance-based tasks. The evidence did not support this. Bayesian regression models suggested moderate evidence for the absence of an association between psychological decentering and (1) sustained attention as measured using the affective SART, (2) working memory recall as measured using the eWMT and (3) cognitive control using the Stroop. This was the case both in affective conditions of the tasks and neutral conditions. It could be the case that the cognitive processes proxied here, such as attention, inhibition and wider executive skills, simply do not relate to psychological decentering. Psychological decentering may instead be associated with a different set of cognitive processes. For example, Bernstein and colleagues have posited a model of decentering that emphasises more self-referential mechanisms, including theory of mind and autobiographical memory, as well as activities such as cognitive re-appraisal (Bernstein et al., 2015). As such, it is important that further studies examine these perspective taking abilities in a comprehensive manner, perhaps by including both psychometric assessment and behavioural tasks (Bennett et al., 2022).

Adolescence is a period characterised by significant social change that can have a long-standing impact on one’s views on, and sense of, self (Choudhury et al., 2006). Thus, we were curious as to whether the ability to adopt a decentered self-perspective, in which one’s own mental experiences are held in awareness, might change the impact of external social influences, such as peers. We, therefore, proposed that higher psychological decentering scores would be associated with reduced susceptibility to peer influence and greater altruistic giving. Our results did not support these hypotheses. This could be evidence against the notion that decentering interacts with constructs such as peer influence in this age group. Indeed, Bayesian regression analysis found moderate-strong evidence for the absence of an effect. We therefore suggest further research to explore the links more thoroughly between psychological decentering in adolescence and factors associated with interpersonal and social change.

As adolescents increased in age from 11 to 19 years, psychological decentering scores decreased. There was no pre-registered hypothesis for this outcome. One explanation for these results could be that adolescence is a period of neuro-developmental “mismatch” – characterised by early maturing sensitivity to affective cues and a later maturing ability to respond using higher-order skills like decentering. This may mean that early and mid-adolescents become more vulnerable to emotional distress, while improvements in their capacity to cope lag. Decentering might therefore decrease in early and mid-adolescent years, as this under-matured skill becomes ill-equipped to manage increased sensitivity to socio-emotional demands. This could then be followed by an increase in capacity during older adolescent and early adulthood as higher-order capacities mature. Evidence suggests, for example, that the capacity to understand and represent mental states – or theory of mind – continues to develop into later adolescent and early adult years (Dumontheil et al., 2010). One possibility therefore is that a larger sample with a wider age range might reveal a non-linear pattern of maturation for psychological decentering across development.

The analysis utilised a secondary dataset, thus the self-report and performance-based measures were not chosen with decentering specifically in mind. This means our ability to investigate cognitive correlates of psychological decentering was limited. As discussed, future research should examine the role of other factors such as theory of mind, perspective taking and cognitive re-appraisal. Another limitation was the absence of a measure of psychological decentering in the dataset. We developed a proxy measure by collating items from different inventories that matched those used in psychological decentering research. Our measure had good internal reliability and construct validity – scores on our measure of decentering correlate with depression and anxiety symptoms as expected. Nonetheless, it would have been preferable to use a gold standard measure of psychological decentering. It is therefore important that future research replicate these results with an established measure of decentering, such as the Experiences Questionnaire (Fresco et al., 2007). The authors are developing such a research line (https://osf.io/9crmp).

A further limitation of these results is the approach used to measure executive function. Each individual component of the task was investigated in regards to its relationship with decentering, rather than taking a latent-variable approach. This approach can mean that results are affected by the “task impurity problem”. As executive functions manifest themselves by interacting with other cognitive processes, all executive tasks strongly implicate other cognitive processes that may not be relevant to the investigated executive function. As such, individual scores on reaction time or error rate may not provide an accurate representation of executive function. In retrospect, a latent-variable approach may have been more effective at elucidating relationships between decentering and executive function, and future research should make use of these techniques (Miyake et al., 2000). A final limitation is the gender ratio of this study, in which 31% of participants were male. This limits generalizability of findings. The research team is conducting further studies to assess whether the results of this study are replicable in a gender-balanced sample.

In conclusion, this study contributes to a burgeoning body of literature on the role of psychological decentering in youth mental health. Results concur with current thinking on associations between decentering and mental health symptoms. However, the cause of association is still unclear, as moderate evidence for the absence of association between decentering and behavioural proxies of executive function was found. Further research is therefore needed to elucidate cognitive correlates of this self-referential capacity and better understand its developmental trajectory. This will be crucial for the development of theoretical accounts of psychological decentering that can guide treatment for youth mental health. Specifically, a fine-grained understanding of the mechanisms underlying psychological decentering could create opportunities to optimise extant psychological therapies that target this capacity and reveal new avenues of interventions to boost it.

## Supplementary Material

Appendices.docx

Supplemental_Materials_AugResubmission clean.docx

## Data Availability

The datasets generated during and/or analysed during the current study will be stored in a publically available repository. Following the publication of the first manuscript, a fully anonymized dataset will be made available online on open-access databases whose servers are located within the UK or Europe. Data processing scripts and programming codes will also be made available online.

## Note

1
Supplemental Bayesian analyses were conducted to test strength of absence of association in the case of non-significant results. Results for these are reported in Supplemental Materials.

## Appendices

### Appendix A. Decentering self-report inventory items

**Table:**

Scale	Item
EQ2	I can observe unpleasant feelings without being drawn into them.
EQ3	I notice that I don’t take difficulties so personally.
EQ7	I can slow my thinking at times of stress.
EQ8	I can actually see that I am not my thoughts.
EQ9	I am consciously aware of a sense of my body as a whole.
EQ11	I view things from a wider perspective.
DDS4	Anxious thoughts. Things have not been going well at school or at your job, and work just keeps piling up. To what extent would you normally be able to defuse from anxious thoughts like “I’ll never get this done”.?
DDS5	Thoughts of self. Imagine you are having a thought such as “no one likes me”. To what extent would you normally be able to defuse from negative thoughts about yourself?
DDS6	Thoughts of hopelessness. You are feeling sad and stuck in a difficult situation that has no obvious end in sight. You experience thoughts such as “Things will never get any better”. To what extent would you normally be able to defuse from thoughts of hopelessness?
DDS10	Feelings of sadness. Imagine that you lose out on something you really wanted. You have feelings of sadness. To what extent would you normally be able to defuse from feelings of sadness?
TMS-D6	I approached each experience by trying to accept it, no matter whether it was pleasant or unpleasant.
TMS-D7	I was aware of my thoughts and feelings without overidentifying with them.
BAFT1	I need to get a handle on my anxiety and fear for me to have the life I want.
BAFT3	I can’t really do the things that I want to do when I have anxiety and fear.
BAFT6	My anxious thoughts and feelings are a problem.
BAFT7	I am sure to be embarrassed and make a fool of myself when other people notice how nervous and shaky I feel
BAFT8	Unusual body sensations are scary and something I need to act on to reduce or get rid of before I can do anything else
BAFT9	My anxious thoughts and feelings are not normal
BAFT12	I could lose control of myself when I feel anxious or afraid.
CFQ1	My thoughts cause me distress or emotional pain
CFQ2	I get so caught up in my thoughts that I am unable to do the things that I most want to
CFQ4	I struggle with my thoughts
CFQ5	I get upset with myself for having certain thoughts
CFQ6	I tend to get very entangled in my thoughts
CFQ7	It’s such a struggle to let go of upsetting thoughts even when I know that letting go would be helpful

### Appendix B. Decentering items and matched candidate items

**Table:**

Table 2	Items from Naragon-Gainey et al. and Matched Items from the CAMM and DERS		
Scale	Item	Candidate Scale	Candidate Item
BAFT3	I can’t do the things that I want when I have anxiety/fear	CAMM1	I get upset with myself for having feelings that don’t make sense.
BAFT6	My anxious thoughts/feelings are a problem	CAMM10	I stop myself from having feelings that I don’t like.
BAFT7	I feel embarrassed when people notice how nervous I feel	CAMM2	At school, I walk from class to class without noticing what I’m doing
BAFT8	Unusual body sensations are scary and need to be reduced	CAMM5	I push away thoughts that I don’t like.
BAFT9	My anxious thoughts and feelings are not normal	CAMM7	I get upset with myself for having certain thoughts
BAFT12	I could lose control of myself when I feel anxious or afraid	CAMM1	I get upset with myself for having feelings that don’t make sense.
CFQ1	My thoughts cause me distress or emotional pain	CAMM9	I think that some of my feelings are bad and that I shouldn’t have them.
CFQ2	I get so caught up in my thoughts that I can’t do things	DERS10	When I’m upset, I acknowledge my emotions.
CFQ4	I struggle with my thoughts	DERS12	When I’m upset, I become embarrassed for feeling that way.
CFQ5	I get upset with myself for having certain thoughts	DERS15	When I’m upset, I believe that I will remain that way for a long time.
CFQ6	I tend to get very entangled in my thoughts	DERS20	When I’m upset, I can still get things done.
CFQ7	It’s such a struggle to let go of upsetting thoughts even when I know that letting go would be helpful	DERS20	When I’m upset, I can still get things done.
DDS5	I can defuse from negative thoughts about myself	DERS22	When I’m upset, I know that I can find a way to eventually feel better.
DDS10	I can defuse from feelings of sadness	DERS21	When I’m upset, I feel ashamed at myself for feeling that way.
EQ2	I can observe unpleasant feelings without being drawn in	DERS31	When I’m upset, I believe that wallowing in it is all I can do.
EQ3	I don’t take difficulties personally	DERS31	When I’m upset, I believe that wallowing in it is all I can do.
EQ7	I can slow my thinking during stress	DERS32	When I'm upset, I lose control over my behaviour
EQ8	I can see that I am not my thoughts	DERS33	When I’m upset, I have difficulty thinking about anything else.
EQ9	I have a sense of my body as a whole	DERS36	When I’m upset, my emotions feel overwhelming.
EQ11	I can view things from wider perspective	DERS30	When I’m upset, I start to feel very bad about myself.
TMS-D7	I am aware of my thoughts/feelings without overidentifying with them	DERS36	When I’m upset, my emotions feel overwhelming.

### Appendix C. Finalised decentering scale.

**Table:**

Final self-rated decentering measure	
1	I get upset with myself for having feelings that don’t make sense.
2	At school, I walk from class to class without noticing what I’m doing
3*	I push away thoughts that I don’t like.
4*	I get upset with myself for having certain thoughts
5*	I think that some of my feelings are bad and that I shouldn’t have them.
6*	I stop myself from having feelings that I don’t like.
7	When I’m upset, start to feel very bad I myself.
8	When I’m upset, I believe that wallowing in it is all I can do.
9	When I'm upset, I lose control over my behaviour
10	When I’m upset, I have difficulty thinking about anything else.
11	When I’m upset, my emotions feel overwhelming.
An asterisk marks the items removed for the 7-item version of the scale.	
