# Supplementary material for: Investigation of the mental health and cognitive correlates of psychological decentering in adolescence
Source: Cogn Emot. 2024 Oct 2;39(2):465–75. doi: 10.1080/02699931.2024.2402947 (PMC11875431; doi:10.1080/02699931.2024.2402947)
Supplement: Appendices.docx [file PCEM_A_2402947_SM4034.docx]

**Appendix A – Decentering Self Report Inventory Items**

| Scale | Item |
| --- | --- |
| EQ2 | I can observe unpleasant feelings without being drawn into them. |
| EQ3 | I notice that I don’t take difficulties so personally. |
| EQ7 | I can slow my thinking at times of stress. |
| EQ8 | I can actually see that I am not my thoughts. |
| EQ9 | I am consciously aware of a sense of my body as a whole. |
| EQ11 | I view things from a wider perspective. |
| DDS4 | Anxious thoughts. Things have not been going well at school or at your job, and work just keeps piling up. To what extent would you normally be able to defuse from anxious thoughts like ‘‘I’ll never get this done.’’? |
| DDS5 | Thoughts of self. Imagine you are having a thought such as ‘‘no one likes me.’’ To what extent would you normally be able to defuse from negative thoughts about yourself? |
| DDS6 | Thoughts of hopelessness. You are feeling sad and stuck in a difficult situation that has no obvious end in sight. You experience thoughts such as ‘‘Things will never get any better.’’ To what extent would you normally be able to defuse from thoughts of hopelessness? |
| DDS10 | Feelings of sadness. Imagine that you lose out on something you really wanted. You have feelings of sadness. To what extent would you normally be able to defuse from feelings of sadness? |
| TMS-D6 | I approached each experience by trying to accept it, no matter whether it was pleasant or unpleasant. |
| TMS-D7 | I was aware of my thoughts and feelings without overidentifying with them. |
| BAFT1 | I need to get a handle on my anxiety and fear for me to have the life I want. |
| BAFT3 | I can’t really do the things that I want to do when I have anxiety and fear. |
| BAFT6 | My anxious thoughts and feelings are a problem. |
| BAFT7 | I am sure to be embarrassed and make a fool of myself when other people notice how nervous and shaky I feel |
| BAFT8 | Unusual body sensations are scary and something I need to act on to reduce or get rid of before I can do anything else |
| BAFT9 | My anxious thoughts and feelings are not normal |
| BAFT12 | I could lose control of myself when I feel anxious or afraid. |
| CFQ1 | My thoughts cause me distress or emotional pain |
| CFQ2 | I get so caught up in my thoughts that I am unable to do the things that I most want to |
| CFQ4 | I struggle with my thoughts |
| CFQ5 | I get upset with myself for having certain thoughts |
| CFQ6 | I tend to get very entangled in my thoughts |
| CFQ7 | It’s such a struggle to let go of upsetting thoughts even when I know that letting go would be helpful |

**Appendix B – Decentering Items and Matched Candidate Items**

| **Table 2** | **Items from Naragon-Gainey et al. (2017) and Matched Items from the CAMM and DERS** |  |  |
| --- | --- | --- | --- |
| Scale | Item | Candidate Scale | Candidate Item |
| BAFT3 | I can’t do the things that I want when I have anxiety/fear | CAMM1 | I get upset with myself for having feelings that don’t make sense. |
| BAFT6 | My anxious thoughts/feelings are a problem | CAMM10 | I stop myself from having feelings that I don’t like. |
| BAFT7 | I feel embarrassed when people notice how nervous I feel | CAMM2 | At school, I walk from class to class without noticing what I’m doing |
| BAFT8 | Unusual body sensations are scary and need to be reduced | CAMM5 | I push away thoughts that I don’t like. |
| BAFT9 | My anxious thoughts and feelings are not normal | CAMM7 | I get upset with myself for having certain thoughts |
| BAFT12 | I could lose control of myself when I feel anxious or afraid | CAMM1 | I get upset with myself for having feelings that don’t make sense. |
| CFQ1 | My thoughts cause me distress or emotional pain | CAMM9 | I think that some of my feelings are bad and that I shouldn’t have them. |
| CFQ2 | I get so caught up in my thoughts that I can’t do things | DERS10 | When I’m upset, I acknowledge my emotions. |
| CFQ4 | I struggle with my thoughts | DERS12 | When I’m upset, I become embarrassed for feeling that way. |
| CFQ5 | I get upset with myself for having certain thoughts | DERS15 | When I’m upset, I believe that I will remain that way for a long time. |
| CFQ6 | I tend to get very entangled in my thoughts | DERS20 | When I’m upset, I can still get things done. |
| CFQ7 | It’s such a struggle to let go of upsetting thoughts even when I know that letting go would be helpful | DERS20 | When I’m upset, I can still get things done. |
| DDS5 | I can defuse from negative thoughts about myself | DERS22 | When I’m upset, I know that I can find a way to eventually feel better. |
| DDS10 | I can defuse from feelings of sadness | DERS21 | When I’m upset, I feel ashamed at myself for feeling that way. |
| EQ2 | I can observe unpleasant feelings without being drawn in | DERS31 | When I’m upset, I believe that wallowing in it is all I can do. |
| EQ3 | I don’t take difficulties personally | DERS31 | When I’m upset, I believe that wallowing in it is all I can do. |
| EQ7 | I can slow my thinking during stress | DERS32 | When I'm upset, I lose control over my behaviour |
| EQ8 | I can see that I am not my thoughts | DERS33 | When I’m upset, I have difficulty thinking about anything else. |
| EQ9 | I have a sense of my body as a whole | DERS36 | When I’m upset, my emotions feel overwhelming. |
| EQ11 | I can view things from wider perspective | DERS30 | When I’m upset, I start to feel very bad about myself. |
| TMS-D7 | I am aware of my thoughts/feelings without overidentifying with them | DERS36 | When I’m upset, my emotions feel overwhelming. |

**Appendix C – Finalised Decentering Scale**

| **Final Self-Rated Decentering Measure** | |
| --- | --- |
| 1 | I get upset with myself for having feelings that don’t make sense. |
| 2 | At school, I walk from class to class without noticing what I’m doing |
| 3* | I push away thoughts that I don’t like. |
| 4* | I get upset with myself for having certain thoughts |
| 5* | I think that some of my feelings are bad and that I shouldn’t have them. |
| 6* | I stop myself from having feelings that I don’t like. |
| 7 | When I’m upset, I start to feel very bad about myself. |
| 8 | When I’m upset, I believe that wallowing in it is all I can do. |
| 9 | When I'm upset, I lose control over my behaviour |
| 10 | When I’m upset, I have difficulty thinking about anything else. |
| 11 | When I’m upset, my emotions feel overwhelming. |
| An asterisk marks the items removed for the 7-item version of the scale. | |
