# Supplementary material for: Investigation of the mental health and cognitive correlates of psychological decentering in adolescence
Source: Cogn Emot. 2024 Oct 2;39(2):465–75. doi: 10.1080/02699931.2024.2402947 (PMC11875431; doi:10.1080/02699931.2024.2402947)
Supplement: Supplemental_Materials_AugResubmission clean.docx [file PCEM_A_2402947_SM4033.docx]

**Supplemental Materials**

**Further Trial Details**

Participants were randomly allocated to two groups: the active condition that involved a mindfulness training programme named .b (pronounced dot-be), or the control condition that involved learning academic study skills. Each programme ran once per week for eight weeks, and participants completed a range of questionnaires both directly before (Time 1) and after (Time 2) participation. Participants also received follow-up assessment after 6 months (Time 3). The data used in this study was from Time 1.

Primary mental health outcomes for the MYRIAD study included measures of depression (Centre for Epidemiological Studies – Depression Scale, CES-D; (Radloff, 1977)) everyday conduct, emotional symptoms, hyperactivity, peer difficulties and pro-social tendencies subscales (Strengths and Difficulties Questionnaire, SDQ; (Goodman, 2001), and a wellbeing measure (Warwick-Edinburgh Mental Wellbeing Scale, WEMWBS; (Tennant et al., 2007)). Secondary mental health measures included an anxiety scale (Revised Child Anxiety and Depression Scale – Short Version, RCADS; (Ebesutani et al., 2012)), a mindfulness scale (Child-Adolescent Mindfulness Measure, CAMM; (Greco et al., 2011)), an emotion regulation scale (Difficulties in Emotion Regulation Scale, DERS; (Gratz & Roemer, 2003)), rumination measure (Ruminative Response Scale, RRS; (Nolen-Hoeksema, 2000), and loneliness (UCLA loneliness scale, UCLALS, (Russell et al., 1980)).

**Full Measure Creation Protocol**

The MYRIAD dataset did not include a self-report inventory of decentering. Therefore, a scale was developed using available items from two other emotional regulation questionnaires - the Difficulties in Emotion Regulation Scale (DERS) and the Child and Adolescent Mindfulness Measure (CAMM). To create this scale, items from decentering self-report inventories were matched to items in the DERS and the CAMM – hereon referred to as “candidate items”.

A previous exploratory factor analysis of five current decentering self-report inventories (total 51 items) by Naragon-Gainey et al. retained 25 items – available in Appendix A - that significantly contributed to decentering scores (Naragon-Gainey & DeMarree, 2017). These items were then compared against candidate items (by MB and RK independently and then assessed for inter-rater agreement) to ascertain if the candidate items were judged to closely resemble any of the existing decentering items from the Naragon-Gainey et al study. Twenty-one candidate items were matched to decentering items using this protocol. The original decentering items and the matching candidate items can be found in the Appendix B.

The similarity between the original decentering items and candidate items was then independently rated by a team of doctoral and post-doctoral level researchers in psychology (N=15). The similarity of the two items was rated on a 5-point Likert scale, from 1 meaning ‘not similar at all’, and 5 meaning ‘indistinguishable’. Matching pairs were excluded if the highest percentage of researchers rated an item as "Not similar at all". Four items were removed for this reason. An initial reliability analysis was then conducted using SPSS. Item-total statistics indicated an increased Cronbach’s alpha if 6 items were removed, and the 6 items were thus removed (George & Mallery, 2003). These steps resulted in the retention of 11 decentering items.

All decentering items were scored on a 5 point Likert scale from 1 to 5. The items were reviewed to ensure all were scored in a manner in which a higher score indicated higher self-reported ability to decenter. As such, items that were not congruent with this scoring were reverse scored. Finally, the item scores were added together to create a total score per participant. This score indicates participants’ self-rated trait ability to decenter. This gave an 11 item scale for which scores ranged from 11-55. The final version is available in Appendix C. The scale had good reliability (Cronbach’s α = .87).

## Normality Testing & Data Transformation

Preliminary normality testing was conducted to assess whether variables met assumptions necessary for regression models. Shapiro Wilks tests indicated significant evidence for lack of normal distribution in residuals for all independent variables, with statistics reported in Supplemental Table 1. Decentering, depression symptoms, and anxious symptoms were significantly skewed, and thus were square or square-root transformed prior to analysis, depending on direction of skew. Details of skewness prior to and post-transformation can be found in Supplemental Table 1. Assumptions of multi-collinearity were met, details of which can also be found in Supplemental Table 1.

| **Supplemental Table 1 - Normality Testing of Independent Variables** | | | | | |
| --- | --- | --- | --- | --- | --- |
|  | Shapiro-Wilks Normality Test | | Skewness | | Multi-Collinearity |
|  | W | *p* | Pre | Post | VIF |
| Self-Rated Decentering | 0.972 | 0.000* | -0.528 | 0.005 | 1.095 |
| Depression (CES-D) | 0.937 | 0.000* | 1.012 | 0.078 | 2.913 |
| Anxiety (RCADS) | 0.944 | 0.000* | 0.918 | 0.042 | 1.723 |
| Wellbeing (WEMWBS) | 0.992 | 0.006* | -0.124^#^ | - | 2.261 |
| *P* values marked with an * indicate significance (*p <* 0.001).  ^#^ indicates that the variable did not meet threshold for transformation (+/- 0.5).  VIF = Variance Inflation Factor (threshold for violation of assumption is 10) | | | | | |

**Bayesian Modelling of Associations between Decentering and Affective Executive Control**

It was unclear whether this finding reflected the presence of statistical error, or instead an absence of an association between these variables. Therefore, and as robustness check, Bayesian Linear Regressions were calculated as alternatives to test strength of evidence for absence of association. This analysis strategy was supplemental. All models showed moderate evidence of absence of effect of decentering in predicting affective cognitive control (BF_M_ > 0.33 for each model). (Supplemental Table 2).

A Bayesian linear model was calculated to test whether social influence scores or prosocial giving scores were predicted by decentering scores (Supplemental Table 2). There was moderate evidence in favour of an absence of effect of decentering in predicting social influence scores (BF_M_ > 0.33), and strong evidence in favour of an absence of effect of decentering in predicting prosocial giving scores (BF_M_ > 0.1).

| **Supp. Table 2 – Bayesian Regression Models Predicting Task Scores Using Decentering Scores** | | | | | | |
| --- | --- | --- | --- | --- | --- | --- |
| Task | Model | P(M) | P(M\|data) | BF_M_ | BF_10_ | R_2_ |
| Social Influence | Null Model | 0.500 | 0.898 | 8.767 | 1.000 | 0.000 |
|  | Decentering * Absolute Rating Difference | 0.500 | 0.102 | 0.114 | 0.114 | 0.000 |
| Prosocial Giving | Null Model | 0.500 | 0.914 | 10.563 | 1.000 | 0.000 |
|  | Decentering * Percentage Donated | 0.500 | 0.086 | 0.095 | 0.095 | 0.000 |
| eWMT | Null Model | 0.500 | 0.870 | 6.666 | 1.000 | 0.000 |
|  | Decentering * eWM Index | 0.500 | 0.130 | 0.150 | 0.150 | 0.002 |
| aSART | Null Model | 0.500 | 0.818 | 4.484 | 1.000 | 0.000 |
|  | Decentering * Commission Errors | 0.500 | 0.182 | 0.223 | 0.223 | 0.003 |
| aSART | Null Model | 0.500 | 0.834 | 5.012 | 1.000 | 0.000 |
|  | Decentering * Reaction Time Variance | 0.500 | 0.166 | 0.200 | 0.200 | 0.003 |
| Stroop | Null Model | 0.500 | 0.889 | 7.985 | 1.000 | 0.000 |
|  | Decentering * Negative and Neutral Diff | 0.500 | 0.111 | 0.125 | 0.125 | 0.001 |
| Stroop | Null Model | 0.500 | 0.843 | 5.362 | 1.000 | 0.000 |
|  | Decentering * Positive and Neutral Diff | 0.500 | 0.157 | 0.187 | 0.187 | 0.003 |
| Stroop | Null Model | 0.500 | 0.904 | 9.465 | 1.000 | 0.000 |
|  | Decentering * Positive and Negative Diff | 0.500 | 0.096 | 0.106 | 0.106 | 0.000 |

**Decentering and Cognitive Bias**

**Rationale**

A number of exploratory analyses were pre-registered to investigate the relationship between decentering and aspects of cognitive bias, using two available measures from the existing dataset. Full details of this task are available at (Dunning et al., 2022). These were as follows:

**Delay Discounting Task.** This task assessed participants on their ability to resist a smaller immediate reward in favour of a larger reward which required them to wait. The participants were presented with a series of two options, for example receiving £10 today or £20 in two weeks’ time, and then chose which they would prefer. The dependent variable was the proportion of delayed reward choices relative to immediate reward choices. This was calculated as a percentage.

**Sunk-Cost Bias Task.** This task assessed the tendency to persist with investment (financial, temporal or other) despite the fact that the costs could not be recovered and that it would no longer be beneficial to continue. This is often referred to as the sunk-cost bias (Mankiw, 2011).This task presented participants with scenarios involving a sunk cost, either financial or time/effort related, and asked them to indicate how they would respond to the situation on a Likert scale (e.g. most likely to do X or most likely to do Y, where Y represented resisting the sunk cost bias). The dependent variable was participants’ average rating, with a higher rating showing greater resistance to bias.

**Results**

Means and standard deviations of variables from both the sunk cost bias task and delay discounting task are available in Supplemental Table 3. Results of regression models investigating the relationship between decentering and each cognitive bias task variable are available in Supplemental Table 4. None of the regression models reached significance.

Due to the absence of significant models, a further Bayesian regression was executed to investigate the level of evidence for an absence of an effect. Results for these analyses can be found in Supplemental Table 5. There was moderate evidence in favour of an absence of effect of decentering in predicting sunk cost bias and delay discounting scores (BF_M_ > 0.33).

| **Supp. Table 3 - Means and Standard Deviations of Cognitive Bias Task Data** | *M* | *SD* |
| --- | --- | --- |
| Delay Discounting Task | 0.430 | 0.166 |
| Sunk Cost Task | 13.52 | 2.859 |

| **Supp. Table 4 – Linear Regression Models Predicting Task Scores Using Decentering** | | | | | | | | | | |
| --- | --- | --- | --- | --- | --- | --- | --- | --- | --- | --- |
| Task | | Model | | | *R^2^* | | *F* | | *p* | *f^2^* |
| Delay Discounting | | Decentering * Delay Discounting Percentage | | | -0.001 | | 0.524 | | 0.469 | -0.00 |
| Sunk Cost Task | | Decentering * Sunk Cost Sum | | | -0.001 | | 0.524 | | 0.469 | -0.00 |
| *df = 1,551* | | | | | | | | | | |
| **Supp. Table 5 – Bayesian Regression Models Predicting Task Scores Using Decentering** | | | | | | | | | | |
| Task | Model | | P(M) | P(M\|data) | | BF_M_ | | BF_10_ | | R_2_ |
| Delay Discounting | Null Model | | 0.500 | 0.891 | | 8.196 | | 1.000 | | 0.000 |
|  | Decentering * Delay Discounting Percentage | | 0.500 | 0.109 | | 0.122 | | 0.122 | | 0.001 |
| Sunk Cost | Null Model | | 0.500 | 0.891 | | 8.194 | | 1.000 | | 0.000 |
|  | Decentering * Sunk Cost Sum | | 0.500 | 0.109 | | 0.122 | | 0.122 | | 0.001 |

**Decentering and Neutral Conditions of Affective Executive Control Tasks**

Exploratory regression models were run to assess the relationship between decentering and executive control in emotionally neutral conditions. Results for these models can be found in Supplementary Table 6. None of these models reached significance.

| **Supp. Table 6 – Linear Regression Models Predicting Neutral Conditions of Affective Tasks Using Decentering Scores** | | | | | |
| --- | --- | --- | --- | --- | --- |
| Task | Model | *R^2^* | *F* | *p* | *f^2^* |
| eWMT | Decentering * eWM Percentage Recall | 0.009 | 5.916 | 0.015* | 0.01 |
| aSART | Decentering * Commission Errors | -0.002 | 0.009 | 0.925 | -0.00 |
|  | Decentering * Reaction Time Variance | 0.001 | 1.826 | 0.177 | 0.00 |
| Stroop Task | Decentering * Neutral Reaction Times | 0.006 | 4.56 | 0.033* | 0.01 |
|  | Decentering * Accuracy on Neutral Trials | 0.010 | 6.37 | 0.012* | 0.01 |
|  | Decentering * RT Variance on Neutral Trials | 0.013 | 8.312 | 0.004* | 0.01 |
| *df = 1,551. * =* statistically significant *p <* 0.05 | | | | | |

**Further Analyses**

**Factor Structure**

A CFA was calculated to check whether the 11 selected items converged on a single factor. Findings indicated a poor fit of a single factor model (χ2 = 531.33, p < .001, CFI = 0.80, TLI = 0.76). An exploratory factor analysis was therefore calculated and first the number of the factors to retain was determined. The scree plot and Kaiser’s rule suggested a two-factor solution (Figure S1a overleaf). Next, an exploratory factor analysis was calculated. This was completed using Goldberg’s bass-ackwards hierarchical method in R (Goldberg, 2006) – an EFA with oblique rotation that also allows factors to be correlated across different hierarchical levels (e.g., Holmes, Mareva, Bennett, Black & Guy, 2021). This EFA suggested that 9-items from both the DERS and the CAMM loaded onto a single factor (C1; r = 0.41 – 0.85) while 4 items from the CAMM only loaded onto a second factor (C2; r = 0.41-0.83) (See Figure S1b overleaf).

Items loading on C1 seemed to reflect heightened identification with one’s mental experiences; that is, a lack of psychological decentering (e.g. “When I’m upset, my emotions feel overwhelming”. Items loading on C2 seemed to reflect the struggle associated with one’s mental experiences; that is, the consequences of a lack of psychological decentering (e.g. “I push away thoughts that I don’t like”). This is somewhat consistent with extant models that define psychological decentering on the basis of (1) a processing of noticing and identifying with mental experiences (C1) and (2) a process of reacting to these mental states (C2). Factor C1 might therefore best reflect the concept of psychological decentering. And the pre-registered analyses were recalculated using these 7-items. Findings are presented in Supplemental Table 7. However, they are entirely consistent with the analysis reported in the main manuscript.

| **Supp. Table 7 – Analysis of Pre-Registered Regression Models with 7-item Scale** | | | | |
| --- | --- | --- | --- | --- |
| Hypothesis | Model | *R^2^* | *F* | *p* |
| 1a | Decentering * Depression | 0.489 | 176.70 | <0.001 |
|  | Decentering * Anxiety | 0.447 | 149.20 | <0.001 |
| 1b | Decentering * Wellbeing | 0.314 | 85.03 | <0.001 |
| 2 | Decentering * eWM Index | 0.001 | 0.63 | 0.425 |
| 3 | Decentering * Commission Errors | 0.000 | 0.20 | 0.659 |
|  | Decentering * Reaction Time Variance | 0.005 | 2.92 | 0.088 |
| 4 | Decentering * Negative and Neutral Diff | 0.000 | 0.15 | 0.696 |
|  | Decentering * Positive and Neutral Diff | 0.001 | 0.75 | 0.386 |
|  | Decentering * Positive and Negative Diff | 0.001 | 0.28 | 0.599 |
| 5 | Decentering * Social Influence | 0.003 | 1.51 | 0.220 |
| *df = 1,547*  Age and gender were included in models 1a and 1b as covariates. | | | | |


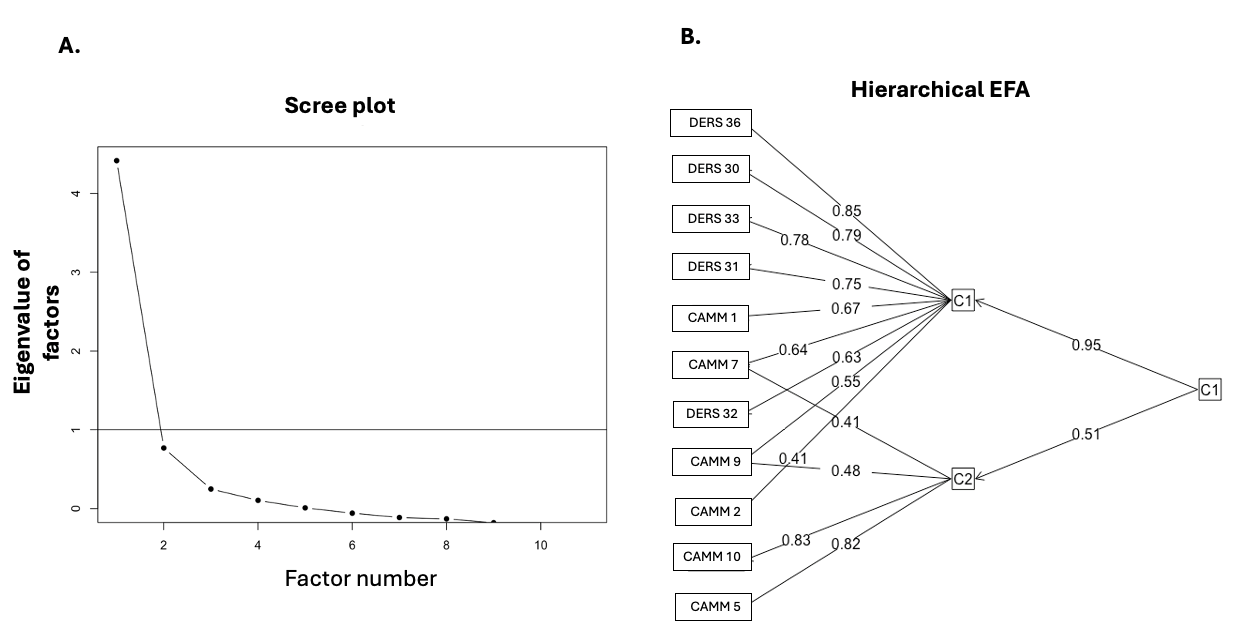


**Supplemental Figure 1a and 1b – Scree Plot and Hierarchical EFA of Decentering Scale**

Bootstrapped Regression Models

To provide further evidence of interpretability and lack of bias in the transformed variable results reported, we fitted regression models to examine each of our hypotheses. This was done in R using the car package. Bootstrapping with 5,000 replications was used to assess the stability of the model estimates. In three models, due to convergence issues in some resampled datasets, between 4313 and 4352 bootstrap replications were completed successfully. This was deemed to be an acceptable number of replications.

The results of the bootstrapped estimates are presented in Supplemental Table 8. These suggested that the model’s estimates are generally robust, with minimal bias. Standard errors for decentering as a predictor in these models were very low, indicating high precision in estimates. Findings are consistent with our main analysis: 95% confidence intervals indicate a significant positive relationship between decentering and wellbeing, as well as a significant negative relationship between decentering and anxiety and depression. 95% Confidence Intervals indicate no association between decentering and our performance-based measures of executive function and social cognition.

| **Supp. Table 8 – Bootstrapped regression models with untransformed variables** | | | | | | | | |
| --- | --- | --- | --- | --- | --- | --- | --- | --- |
| Hyp. | Model | | Original Estimate | Bias | SE | Median | 2.5% | 7.5% |
| 1a | Depression | Intercept | 50.76 | 0.106 | 4.255 | 50.88 | 42.49 | 58.99 |
|  |  | Decentering | -0.789 | -0.001 | 0.049 | -0.791 | -0.877 | -0.685 |
|  |  | Gender$male | -2.006 | -0.009 | 0.631 | -2.006 | -3.22 | -0.784 |
|  |  | Age | -0.099 | -0.004 | 0.207 | -0.103 | -0.510 | 0.320 |
|  | Anxiety | Intercept | 48.57 | 0.021 | 2.849 | 48.62 | 42.65 | 53.97 |
|  |  | Decentering | -0.603 | -0.000 | 0.034 | -0.604 | -0.669 | -0.536 |
|  |  | Gender$male | -1.650 | -0.007 | 0.534 | -1.647 | -2.714 | -0.592 |
|  |  | Age | -0.770 | -0.000 | 0.151 | -0.772 | -1.057 | -0.464 |
| 1b | Wellbeing | Intercept | 24.83 | -0.155 | 4.059 | 24.70 | 17.09 | 33.18 |
|  |  | Decentering | 0.580 | 0.002 | 0.044 | 0.582 | 0.488 | 0.662 |
|  |  | Gender$male | 1.148 | 0.002 | 0.713 | 1.150 | -0.261 | 2.552 |
|  |  | Age | -0.103 | 0.006 | 0.203 | -0.977 | -0.500 | 0.315 |
| 2 | eWM Index | Intercept | -3.755 | 0.030 | 2.337 | -7.734 | -8.354 | 0.680 |
|  |  | Decentering | 0.060 | -0.001 | 0.057 | 0.058 | -0.049 | 0.174 |
| 3 | SART Commission Errors | Intercept | -0.276 | -0.016 | 0.815 | -0.302 | -1.813 | 1.424 |
|  |  | Decentering | 0.018 | 0.000 | 0.019 | 0.018 | -0.023 | 0.054 |
|  | SART Reaction Time Variance | Intercept | -0.005 | 0.000 | 0.017 | -0.005 | -0.039 | 0.029 |
|  |  | Decentering | 0.000 | 0.000 | 0.000 | 0.000 | -0.000 | 0.001 |
| 4 | Negative and Neutral Diff | Intercept | -43.19 | 0.362 | 21.76 | -42.76 | -87.20 | -1.915 |
|  |  | Decentering | 0.330 | -0.010 | 0.535 | 0.319 | -0.692 | 1.399 |
|  | Positive and Neutral Diff | Intercept | -16.28 | 0.224 | 22.55 | -15.63 | -64.23 | 24.54 |
|  |  | Decentering | 0.595 | -0.004 | 0.559 | 0.581 | -0.428 | 1.759 |
|  | Positive and Negative Diff | Intercept | 26.90 | 0.028 | 20.19 | 26.97 | -12.16 | 66.94 |
|  |  | Decentering | 0.264 | 0.001 | 0.505 | 0.264 | -0.742 | 1.261 |
| 5 | Social Influence | Intercept | 0.709 | 0.000 | 0.093 | 0.708 | 0.537 | 0.898 |
|  |  | Decentering | 0.002 | 0.000 | 0.002 | 0.002 | -0.003 | 0.006 |
|  | | | | | | | | |

Supplemental References

Dunning, D., Ahmed, S., Foulkes, L., Griffin, C., Griffiths, K., Leung, J. T., Parker, J., Piera Pi-Sunyer, B., Sakhardande, A., Bennett, M., Haag, C., Montero-Marin, J., Packman, D., Vainre, M., Watson, P., MYRIAD Team, Kuyken, W., Williams, J. M. G., Ukoumunne, O. C., … MYRIAD Team Group. (2022). The impact of mindfulness training in early adolescence on affective executive control, and on later mental health during the COVID-19 pandemic: A randomised controlled trial. *Evidence-Based Mental Health*, *25*(3), 110–116. https://doi.org/10.1136/ebmental-2022-300460

Ebesutani, C., Reise, S. P., Chorpita, B. F., Ale, C., Regan, J., Young, J., Higa-McMillan, C., & Weisz, J. R. (2012). The Revised Child Anxiety and Depression Scale-Short Version: Scale reduction via exploratory bifactor modeling of the broad anxiety factor. *Psychological Assessment*, *24*(4), 833–845. https://doi.org/10.1037/a0027283

George, D., & Mallery, P. (2003). SPSS for Windows Step-by-Step: A Simple Guide and Reference, 14.0 update (7th Edition). *Http://Lst-Iiep.Iiep-Unesco.Org/Cgi-Bin/Wwwi32.Exe/[In=epidoc1.in]/?T2000=026564/(100)*.

Goldberg, L. R. (2006). Doing it all bass-ackwards: The development of hierarchical factor structures from the top down. Journal of Research in Personality, 40(4), 347-358.

Goodman, R. (2001). Psychometric properties of the strengths and difficulties questionnaire. *Journal of the American Academy of Child and Adolescent Psychiatry*, *40*(11), 1337–1345. https://doi.org/10.1097/00004583-200111000-00015

Gratz, K. L., & Roemer, L. (2003). Multidimensional Assessment of Emotion Regulation and Dysregulation: Development, Factor Structure, and Initial Validation of the Difficulties in Emotion Regulation Scale. *Journal of Psychopathology and Behavioral Assessment*, 14.

Greco, L. A., Baer, R. A., & Smith, G. T. (2011). Assessing mindfulness in children and adolescents: Development and validation of the Child and Adolescent Mindfulness Measure (CAMM). *Psychological Assessment*, *23*(3), 606–614. https://doi.org/10.1037/a0022819

Holmes, J., Mareva, S., Bennett, M. P., Black, M. J., & Guy, J. (2021). Higher-order dimensions of psychopathology in a neurodevelopmental transdiagnostic sample. Journal of abnormal psychology, 130(8), 909

Mankiw, N. G. (2011). *Principles of Economics, 5th edition*. South-Western Cengage Learning.

Naragon-Gainey, K., & DeMarree, K. G. (2017). Structure and validity of measures of decentering and defusion. *Psychological Assessment*, *29*(7), 935–954. https://doi.org/10.1037/pas0000405

Nolen-Hoeksema, S. (2000). The role of rumination in depressive disorders and mixed anxiety/depressive symptoms. *Journal of Abnormal Psychology*, *109*(3), 504–511. https://doi.org/10.1037/0021-843X.109.3.504

Radloff, L. S. (1977). The CES-D Scale: A Self-Report Depression Scale for Research in the General Population. *Applied Psychological Measurement*, *1*(3), 385–401. https://doi.org/10.1177/014662167700100306

Russell, D., Peplau, L. A., & Cutrona, C. E. (1980). The revised UCLA Loneliness Scale: Concurrent and discriminant validity evidence. *Journal of Personality and Social Psychology*, *39*(3), 472–480. https://doi.org/10.1037/0022-3514.39.3.472

Tennant, R., Hiller, L., Fishwick, R., Platt, S., Joseph, S., Weich, S., Parkinson, J., Secker, J., & Stewart-Brown, S. (2007). The Warwick-Edinburgh Mental Well-being Scale (WEMWBS): Development and UK validation. *Health and Quality of Life Outcomes*, *5*(1), 63. https://doi.org/10.1186/1477-7525-5-63
